# Image-based mHealth for remote diagnostic assistance a means to promote equity in quality care

**DOI:** 10.1080/16549716.2017.1344004

**Published:** 2017-08-25

**Authors:** Lucie Laflamme

**Affiliations:** ^a^ Department of Public Health Sciences, Global Health, Karolinska Institutet, Stockholm, Sweden; ^b^ Stellenbosch Institute for Advanced Study (STIAS), Wallenberg Research Centre, Stellenbosch University, Stellenbosch, South Africa; ^c^ University of South Africa, Institute for Social and Health Sciences, Johannesburg, South Africa

Smartphones are becoming widely available and increasingly versatile. Within the foreseeable future wireless networks will also be accessible worldwide, thereby removing a major obstacle to smartphones reaching their full performance capacity. Meanwhile, advances on both fronts have sparked the creation of many digital approaches in a wide variety of services. Indeed, applications, apps, are becoming a routine way for many consumers to access information or to deal with their private and professional commitments and responsibilities.

In the field of global health, technologies can be instrumental for a wide range of interventions addressing, for example, malnutrition, sanitation, road safety, or health. Health technologies for their part can be designed to either prevent, diagnose, or treat illness [1]; they can be specific, as in the case of a vaccine for a particular disease, or more widely applicable, e.g. a blood-pressure monitor. mHealth interventions as a specific kind of health technology appeared in the early 2000s and have evolved since then regarding their purpose, the types of mobile devices used for delivery, as well as the health conditions addressed [2]. Interventions based on the use of smart devices (smartphones, tablet PCs and iPod touches), although very recent, represent by far the bulk of those mHealth interventions reported in the scientific literature, and non-communicable conditions are the predominant target [2]. In recent years research reports on mHealth interventions dealing with infectious diseases and maternal and child health have been on the increase.

One untapped domain of application of health apps is diagnostic and treatment support to clinicians, an area where the need is immense in health care delivery globally and the potential for cost-saving tremendous. One example is the conception of user-friendly, image-based mHealth consultation platforms for frontline clinicians where procedures already in place within more traditional forms of telemedicine (e.g. dermatology and radiology) could be modernized and scaled up. Another example is the revolutionary transformation of medical fields relying on the laboratory environment (e.g. pathology or ophthalmology) by providing them with extra-laboratory microscopy assistance (‘lab-on-a-chip’). A third one is that of deep learning whereby computers can be trained to provide health-care professionals with clinical advice and send images to them through smartphone applications.

In sum, image-based diagnostic support through mHealth could significantly increase the readiness of health-care services to deal with wide-ranging global population health threats, both old (e.g. malaria, TB and HIV) and new (e.g. non-communicable diseases in general or trauma in particular) [1]. The scope for applications of this kind regarding cost and life savings is huge since early and accurate diagnosis is key for adequate utilization of resources (and the reduction of unnecessary referrals) and also for better patient outcomes. Additional benefits are the reduction of professional isolation and better recruitment and retention of staff in remote areas. So there is a great potential for more equitable systems in global health care through large-scale support in resource-scarce settings. This is relevant for not only low- and middle-income countries but even high-income countries (HICs).

Certain barriers may contribute to explaining the lack of penetration of solutions of this kind in health-care services to date [3]. One is a poor acceptance by frontline clinicians who have to deal with unforeseen implementation issues that disturb their workflow, detract from time spent with patients and hinder interoperability, with all the frustration that this involves. Health-care providers will definitely be sceptical about the usefulness of mHealth tools if they are not interoperable to the extent that data generated from them are not compatible and cannot be integrated with other clinical information and made accessible through electronic health records. An additional barrier is the actual lack of high-quality evidence of the efficacy of the diagnostic and treatment tools, something that both providers and sponsors can legitimately question. Reports from other domains of application reveal that many mHealth projects that start promisingly often remain at the pilot stage or are not sustained [2]. The technologies are hence not used to their full potential, impeding successful implementation, sustainability and expansion. A third barrier, derived in part from the latter, is that the evidence base that could spark greater interest among the providers is too poor. Therefore, they remain unclear about, on the one hand, the effectiveness and safety of the digital interventions and, on the other hand, how interventions should be implemented and funded.

This special issue is dedicated to image-based mHealth as a tool for diagnostic support and treatment advice. It includes ten contributions. First, four articles address how far mHealth solutions can take us in levels of sophistication and according to which technical, organizational and user conditions. First Boman and Krusse [4] share their view on the contribution of information and communications technology (ICT) in supporting global health goals as conditional to the fulfilment of four kinds of access – to the Internet, either to individual data that describe their actual health, to individual attributes that can be indirectly health-related, or to health-related attributes of the individual environment. Then Lundin and Dumont [5] present image- and sensor-based mHealth solutions with strong potential for diagnostic support in resource-poor settings and highlight elements that are key to making technology and low-cost innovations sustainable and scalable. Focusing on issues related to implementation and scale up, Fölster [6] proposes four criteria that may help determining the extent to which a mHealth solution can ‘go viral’: zero costs to users, healthcare provider being able to recoup costs, being able to handle and increase the demand, and the conception of an mHealth app that does not duplicate development efforts. For their part, Barkman and Weinehall [7] discuss the need for infrastructure and regulatory frameworks, and the necessary involvement of different stakeholders and decision-makers in the process of implementing mHealth solutions to achieve good results. They provide examples from the experience of three countries: Ethiopia, Ghana and Sweden.

Two articles then present clinical perspectives on the implementation and use of mHealth. Having as an entry point the health-care system as a whole, Wallis et al. [8] sketch the regulatory, technological and user perspectives that need to be taken into consideration for mHealth solutions to be adequately integrated into existing health-care systems. Thereafter, with a focus on patient issues and shared decision-making (SDM), Rahimi et al. [9] discuss a number of ‘promises and perils’ pertaining to mHealth for SDM and they also outline a research agenda for the field.

Two subsequent research articles are based on implementations of mHealth interventions for diagnostic support of two global health issues: acute burn injuries and malaria infection. Hasselberg et al. [10] expose some challenges related to the evaluation of image-based mHealth interventions and they discuss ethical and methodological issues in the determination of suitable evaluation designs and pertinent outcome measures in emergency-care settings. In a proof-of-concept study, Holmström et al. [11] take us to the field of digital microscopy and deep learning. They show that parasite identification by visual analysis of digital slides captured by a mobile microscope is feasible for a range of parasites, and that deep-learning-based image analysis can be utilized for the automated detection and classification of helminths.

Wallis et al. [12] present a roadmap for the implementation of mHealth solutions for diagnostic assistance at point of care. The roadmap itself is informed by the contributions of a variety of stakeholders, including researchers, health-care providers, policy-makers and developers from about 15 different countries, all gathered for a two-day round table that took place in Stellenbosch, South Africa, in February 2017. The round table was organized so as to provide a forum for a variety of stakeholders to discuss in broad terms the possibilities that current developments in image-based mHealth offer for timely, accurate and equitable health-care delivery and the challenges that their development and implementation may entail for all potential users/beneficiaries. A diversity of aspects highly relevant to the layout of the roadmap were gradually unfurled in six consecutive sessions, using earlier versions of some of the papers presented in this special issue. The discussions made it clear that the successful implementation of the mHealth solutions at stake will necessitate a seamless introduction into routines, adequate technical support and significant added value.

Finally, Gulliksen [13] explains how digitalization changes society and introduces radically new ways of doing things and addressing issue, not least in the field of human-computer interaction (HCI). He provides examples from different regions of the world as to how new HCI has contributed to international development.

With this special issue, we hope to reach out to those stakeholders that locally, regionally or nationally are in a position to influence the choice, implementation and scale-up of mHealth solutions in such a way that significant benefits arise for all users, to the betterment of health and the reduction of the health divide.

## References

[1] Howitt P, Darzi A, Yang G-Z, et al Technologies for global health. Lancet. 2012;380:507–5. 22857974 10.1016/S0140-6736(12)61127-1

[2] Ali EE, Chew L, Yap KY. Evolution and current status of mhealth research: a systematic review. BMJ Innov. 2016;2:33–40.

[3] Eapen ZJ, Mintu P, Turakhia P, et al Defining a mobile health roadmap for cardiovascular health and disease. JAHA. 2016;5:e003119. 27405809 10.1161/JAHA.115.003119 PMC5015362

[4] Boman M, Krusse E Supporting Global Health goals by information and communications technology. Glob Health Action. 2017;10(S3): 1321904. 10.1080/16549716.2017.1321904 PMC5645697 28838300

[5] Lundin J, Dumont G Medical mobile technologies – what is needed for a sustainable and scalable implementation on a global scale? Glob Health Action. 2017;10(S3): 1344046. 10.1080/16549716.2017.1344046 PMC5786339 28838308

[6] Folster S Viral mHealth. Glob Health Action. 2017;10(S3): 1336006. 10.1080/16549716.2017.1336006 PMC5645666 28838304

[7] Barkman C, Weinehall L Policymakers and mHealth: roles and expectations, with observations from Ethiopia, Ghana and Sweden. Glob Health Action. 2017;10(S3): 1337356. 10.1080/16549716.2017.1337356 PMC5645698 28838303

[8] Wallis L, Blessing P, Dalwai M, et al Integrating mHealth at point of care in low and middle income settings: the system perspective. Glob Health Action. 2017;10(S3): 1327686. 10.1080/16549716.2017.1327686 PMC5645717 28838302

[9] Rahimi SA, Menear M, Robitaille H, et al Are mobile health applications useful for supporting shared decision making in diagnostic and treatment decisions? Glob Health Action. 2017;10(S3): 1332259. 10.1080/16549716.2017.1332259 PMC5645724 28838306

[10] Hasselberg MK, Wallis L, Blessing P, et al A smartphone-based consultation system for acute burns -methodological challenges related to follow-up of the system. Glob Health Action. 2017;10(S3): 1328168. 10.1080/16549716.2017.1328168 PMC5645658 28838311

[11] Holmström O, Linder N, Ngasala B, et al Point-of-care mobile digital microscopy and deep learning for the detection of soil-transmitted helminths and Schistosoma haematobium. Glob Health Action. 2017;10(S3): 1337325. 10.1080/16549716.2017.1337325 PMC5645671 28838305

[12] Wallis L, Hasselberg M, Barkman C, et al A roadmap for the implementation of mHealth innovations for image-based diagnostic support in clinical and public health settings: A focus on front-line health workers and health system organizations. Glob Health Action. 2017;10(S3): 1340254. 10.1080/16549716.2017.1340254 PMC5645694 28838310

[13] Gulliksen J Institutionalizing human computer interaction for global health. Glob Health Action. 2017;10(S3): 1344003. 10.1080/16549716.2017.1344003 PMC5645721 28838309

